# Feasibility of Total Variation Noise Reduction Algorithm According to Various MR-Based PET Images in a Simultaneous PET/MR System: A Phantom Study

**DOI:** 10.3390/diagnostics11020319

**Published:** 2021-02-16

**Authors:** Chan-Rok Park, Seong-Hyeon Kang, Young-Jin Lee

**Affiliations:** 1 Department of Radiological Science, Jeonju University, 303, Cheonjam-ro, Wansan-gu, Jeonju-si, Jeollabuk-do 55069, Korea; tigeaglepcr@jj.ac.kr; 2 Department of Radiological Science, Gachon University, 191, Hambakmoero, Yeonsu-gu, Incheon 21936, Korea; tjdgus7345@nate.com

**Keywords:** positron emission tomography (PET)/magnetic resonance (MR), total variation (TV), noise-reduction algorithm, attenuation correction, nuclear medicine

## Abstract

Recently, the total variation (TV) algorithm has been used for noise reduction distribution in degraded nuclear medicine images. To acquire positron emission tomography (PET) to correct the attenuation region in the PET/magnetic resonance (MR) system, the MR Dixon pulse sequence, which is based on controlled aliasing in parallel imaging, results from higher acceleration (CAIPI; MR-AC_Dixon-CAIPI_) and generalized autocalibrating partially parallel acquisition (GRAPPA; MR-AC_Dixon-GRAPPA_) algorithms are used. Therefore, this study aimed to evaluate the image performance of the TV noise reduction algorithm for PET/MR images using the Jaszczak phantom by injecting ^18^F radioisotopes with PET/MR, which is called mMR (Siemens, Germany), compared with conventional noise-reduction techniques such as Wiener and median filters. The contrast-to-noise (CNR) and coefficient of variation (COV) were used for quantitative analysis. Based on the results, PET images with the TV algorithm were improved by approximately 7.6% for CNR and decreased by approximately 20.0% for COV compared with conventional noise-reduction techniques. In particular, the image quality for the MR-AC_Dixon-CAIPI_ PET image was better than that of the MR-AC_Dixon-GRAPPA_ PET image. In conclusion, the TV noise-reduction algorithm is efficient for improving the PET image quality in PET/MR systems.

## 1. Introduction

Medical imaging in nuclear medicine plays an important role in acquiring functional information about patients using positron emission tomography (PET) with various radioisotopes [1,2]. In addition, the development of hybrid scanners, that is, PET/computed tomography (CT) or PET/magnetic resonance (MR), is helpful in obtaining functional and anatomic information simultaneously. Integrated PET/MR images have advantages over PET/CT images with respect to the reduction of radiation exposure and superior soft-tissue imaging [3,4].

Originally, a PET/MR scanner was developed by separating PET and MR based on sequential imaging because the magnetic fields use in MR distort the gamma signal [5]. Furthermore, the MR magnetic field can interrupt the normal operation of the photomultiplier tube (PMT) and cause artifacts related to eddy currents [6,7]. However, integrated PET/MR, which combines both PET and MR scanners, has been achieved by replacing the PMTs with avalanche photodiodes (APDs) [8,9]. In addition, PET and MR scans are available simultaneously. Torigian et al. reported that integrated PET/MR imaging is more powerful than PET, PET/CT, or MRI alone [10].

The principle of gamma-ray detection in PET is that two annihilation radiations produced between positrons and electrons are detected by a scintillation detector [11]. However, signal loss occurs during the process of gamma ray detection because of attenuation or scattering effects. To overcome this problem, attenuation correction (AC) for PET/CT, which is called CT-based PET imaging, is performed by applying a suitable AC coefficient using the Hounsfield unit value at 511 keV [12,13]. In PET/MR, AC is performed by applying various MR pulse sequences. In general, the Dixon pulse sequence is applied to the AC (MR-AC_Dixon_) [14,15,16,17,18,19,20]. The MR-AC_Dixon_ pulse sequence assigns the attenuation coefficient to soft tissue by dividing it into four categories: background, fat, lungs, and soft tissue [14,15]. To improve the image quality of MR-based PET images based on the MR-AC_Dixon_ pulse sequence, the generalized autocalibrating partially parallel acquisition (GRAPPA) algorithm (MR-AC_Dixon-GRAPPA_) is used as the acceleration factor [16]. The MR-AC_Dixon-GRAPPA_ pulse sequence accelerates the acquisition of MR data by acquiring several k-space values and compensating them to approximate values to achieve a short acquisition time [17]. In addition, the MR-AC_Dixon-GRAPPA_ pulse sequence is expanded by applying controlled aliasing in parallel imaging, resulting in a higher acceleration (CAIPIRINHA) algorithm (MR-AC_Dixon-GAIPI_) [18]. The MR-AC_Dixon-GAIPI_ pulse sequence features a high resolution for breath-hold MR imaging because of the shorter acquisition time (9 s/bed) compared with the PET image-based MR-AC_Dixon-GRAPPA_ pulse sequence [19]. Consequently, the AC process using various MR-AC pulse sequences (MR-AC_Dixon-GRAPPA_ and MR-AC_Dixon-GAIPI_) is necessary to improve the PET image quality.

In addition, noise occurs in nuclear medicine images because of a poor photon count [21]. To solve this problem, there is a technique to increase the number of photons by injecting high radioactivity, but the patient receives a lot of radiation exposure. Therefore, many researchers have proposed noise-reduction algorithms without increasing the radiation exposure [22,23,24]. Wiener and median filters are widely used as conventional filters to reduce noise distribution in degraded images. The degree of noise reduction is effective, but a blurring effect is caused by the loss of high-frequency and edge signals in the images [25,26]. To overcome this drawback, a total variation (TV) algorithm that is able to maintain the edge signal and reduce the noise distribution by setting the region of interest for each pixel in the image has been suggested [27,28]. The TV noise reduction algorithm has already been proven using radiologic images from X-rays [29]. In particular, a study by Kang et al. reported that CNR and COV were significantly improved in all three planes compared with the original image when the TV algorithm was applied to a 4D small-animal CT image [27]. In addition, research by Seo K. et al. confirmed that the TV algorithm greatly improved the image characteristics in the noise distribution in the frequency domain in X-ray images [29]. Recently, a study on the applicability of the TV algorithm to confocal laser scanning microscopy images was also conducted, and a quantitative analysis method of noise level for two color channels was also proposed [30]. However, there is little research in the nuclear medicine field, especially PET/MR images, that has been recently developed.

Therefore, the purpose of this study was to confirm the effectiveness of the TV noise-reduction algorithm for PET/MR images. To evaluate the image performance, images were acquired by applying various MR-AC pulse sequences (MR-AC_Dixon-GRAPPA_ and MR-AC_Dixon-GAIPI_) to PET images with a TV noise-reduction algorithm and compared with conventional techniques such as Wiener and median filters.

## 2. Materials and Methods

### 2.1. Experimental Setup

**Figure 1:**
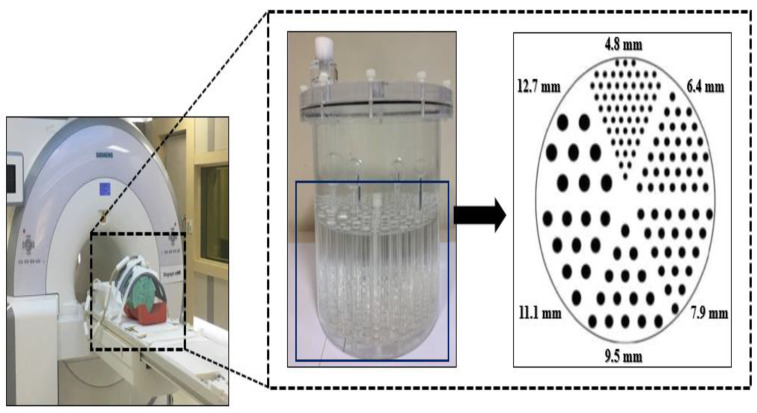
Experimental setup using a positron emission tomography (PET)/magnetic resonance (MR) scanner and a Jaszczak phantom.

The integrated PET/MR hybrid system, which is called mMR (Siemens, München, Germany), was used for the phantom experiments. Figure 1 shows the PET/MR scanner and Jaszczak phantom, which contains rods of various diameters.

**Figure 2:**
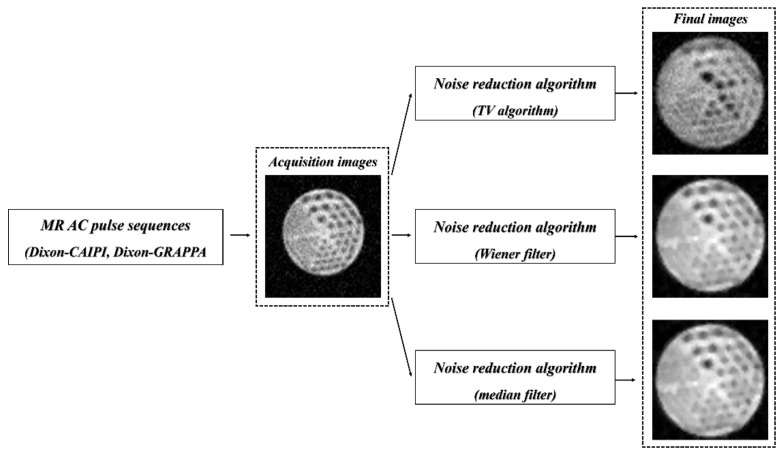
Flowchart for acquisition of images applied to MR_Dixon-CAIPI_ and MR_Dixon-GRAPPA_ AC pulse sequences to final images using the total variation (TV) algorithm, Wiener filter, and median filter for noise reduction.

The detector used lutetium oxyorthosilicate material as a scintillator crystal in PET/MR with an avalanche photodiode array system. To acquire various MR-based PET images, MR_Dixon-CAIPI_ and MR_Dixon-GRAPPA_ pulse sequences were applied to non-AC PET images using a phantom injected with NiSO_4_ + NaCl fluids to acquire more uniform MR-based PET images than MR-based PET images using only water fluid. In general, the water is used as phantom fluid when obtaining the phantom images in nuclear medicine because the radioisotope ^18^F dissolves in water. However, water causes artifacts in MR imaging above 1.5 T due to high relative permittivity [31]. Figure 2 shows a schematic diagram of this study. In this study, the partial-volume effect, which is the loss of apparent activity in small spheres caused by a limited imaging system, is considered using the mMR imaging system.

### 2.2. Modeling of the TV Noise-Reduction Algorithm 

Gaussian noise, which is generated in various medical-imaging systems based on gamma rays and X-rays, should be removed because it degrades the image quality and affects the efficiency of post-processing. To resolve the noise problem, various denoising methods have been proposed, such as local filters, wavelet transform-based techniques, and machine learning. Among these techniques, the TV-based algorithm is known to effectively remove Gaussian noise because it considers the correlation between the signal intensity of the region of interest (ROI) and the overall image configuration variance. The TV algorithm performs a gradient that calculates the difference between the pixel value of the ROI and the surrounding pixel value as follows:

(1) $$\begin{array}{l} ||f{\left(m,n\right)||}_{\mathrm{TV}}=\sum_{m=1}^{M}\sum_{n=1}^{N}\left|\nabla f\left(m,n\right)\right|\\ =\;\sum_{m=1}^{M}\sum_{n=1}^{N}\sqrt{{\left(f\left(m,n\right)-f\left(m-1,n\right)\right)}^{2}+{\left(f\left(m,n\right)-f\left(m,n-1\right)\right)}^{2}} \end{array}$$

where M and N are the numbers of rows and columns in the image $f\left(m,n\right)$, respectively, and ∇ is the gradient operator. The L2-norm has the risk of incorrect image processing, which considers the noise to the edge signal, because it is sensitive to noise, although it is known to be an effective gradient operator for extracting edge signals. For this reason, we designed a gradient operator using the L1-norm. Based on Equation (1), an optimized Rudin-Osher-Fatemi (ROF) model was presented by Rudin et al. [28].

(2) $$\varphi \left[f\left(m,n\right)|I\left(m,n\right)\right]=\overset{M}{\underset{m=1}{\sum }}\overset{N}{\underset{n=1}{\sum }}{\left|\nabla f\left(m,n\right)\right|}_{1}+\frac{\lambda }{2}||I\left(m,n\right)-\;f{\left(m,n\right)||}_{2}$$

where $\frac{\lambda }{2}||I\left(m,n\right)-\;f{\left(m,n\right)||}_{2}$ is the fidelity term indicating the accuracy of the image information, and $\sum_{m=1}^{M}\sum_{n=1}^{N}{\left|\nabla f\left(m,n\right)\right|}_{1}$ is a regularization term that assists the object function (= $\varphi [f\left(m,n\right)|I\left(m,n\right)]$) in finding the correct solution. In addition, λ is a control parameter that balances the two terms, and a value of 0.1 was applied in this study [32,33]. Based on Equation (2), a solution was obtained by iteratively calculating the weight for the pixel of the ROI and its surroundings. To compare the MR-based PET images in PET/MR with application of the TV algorithm, Wiener and median filters were used for correction of noisy images.

### 2.3. Quantitative Analysis

To evaluate image quality, the contrast-to-noise ratio (CNR) and coefficient of variation (COV) were used by drawing ROIs in images with noise, Wiener, median, and TV algorithms. The CNR and COV values were calculated as follows.

(3) $$\mathrm{CNR}=\;\frac{\left|H_{s}-{\;H}_{B}\right|}{\sqrt{\sigma_{s}^{2}+\sigma_{B}^{2}}}$$

(4) $$\mathrm{COV}=\;\frac{\sigma_{s}}{H_{s}}\;$$

where $H_{s}$ and $\sigma_{S}$ are the mean count and standard deviation of the sphere ROI, respectively, and $H_{B}$ and $\sigma_{B}$ are the mean count and standard deviation for the background ROI, respectively. The quantitative analysis method for COV is widely used to confirm noise distribution. In addition, the CNR is used to compare contrast between the sphere and background. For reproducibility analysis, we performed the experiment 10 times under the same conditions.

## 3. Results and Discussion

The PET images based on the MR pulse sequences were developed using an integrated PET/MR system, and there have been many studies that attempted to improve PET image quality [34,35]. In previous studies, we confirmed the usefulness of the TV algorithm for noise reduction compared with conventional noise-reduction methods such as Wiener and median filters in X-ray based images and MR images [36,37]. The results of previous studies show that the TV algorithm not only resulted in efficient noise reduction, but also improvement in signal and contrast in medical images. Based on our previous studies, we evaluated the feasibility of the TV algorithm for noise reduction in MR-AC_Dixon-CAIPI_- and MR-AC_Dixon-GRAPPA_-based PET images using phantom using ^18^F radioisotopes. First, MR-AC PET images were acquired using MR-AC_Dixon-CAIPI_ and MR-AC_Dixon-GRAPPA_ pulse sequences. Subsequently, Gaussian noise with a 0.001 variance parameter was applied to acquire the PET images using MATLAB software. To evaluate the image performance for noise reduction, the proposed TV algorithm was used for noise reduction and compared with conventional noise reduction filters.

**Figure 3:**
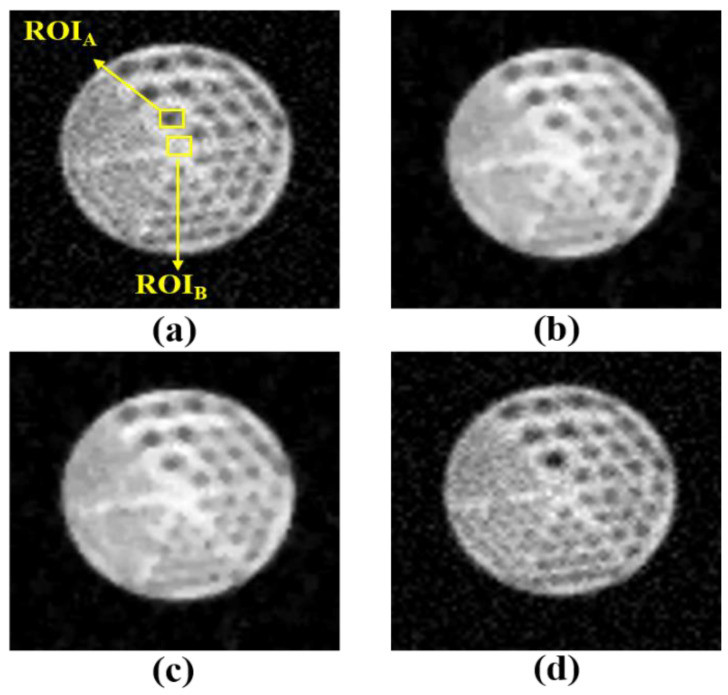
MR-AC_Dixon-CAIPI_-based PET phantom images with (**a**) noise, (**b**) Wiener filter, (**c**) median filter, and (**d**) TV algorithm. The contrast-to-noise ratio (CNR) was calculated using the ROI_A_ and ROI_B_, and the coefficient of variation (COV) was calculated using the ROI_A_.

**Figure 4:**
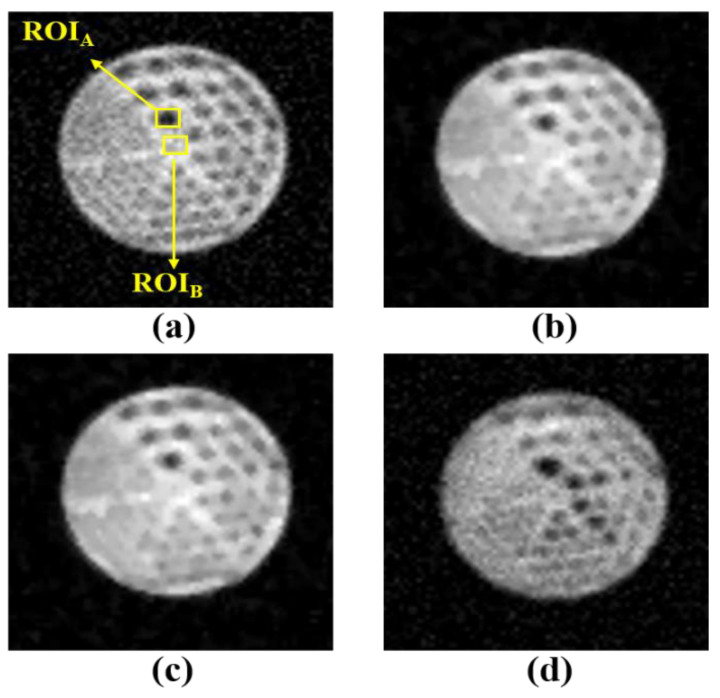
MR-AC_Dixon-GRAPPA_-based PET phantom images with (**a**) noise, (**b**) Wiener filter, (**c**) median filter, and (**d**) TV algorithm. The contrast-to-noise ratio (CNR) was calculated using the ROI_A_ and ROI_B_, and the coefficient of variation (COV) was calculated using the ROI_A_.

Figure 3 and Figure 4 show the MR-AC_Dixon-CAIPI_- and MR-AC_Dixon-GRAPPA_-based PET images according to the noise, Wiener filter, median filter, and TV algorithm, respectively. For the visual evaluation, the PET images with Wiener and median filters as conventional noise-reduction methods assessed the blurring effect in the MR-AC_Dixon-CAIPI_- and MR-AC_Dixon-GRAPPA_-based PET images. Although Wiener and median filters are simple methods to reduce the noise distribution in the spatial domain, there are disadvantages of removing detailed regions and loss of edge signals. However, PET images with a TV algorithm based on a regularization term for noise reduction can distinguish each rod better than conventional filters.

**Figure 5:**
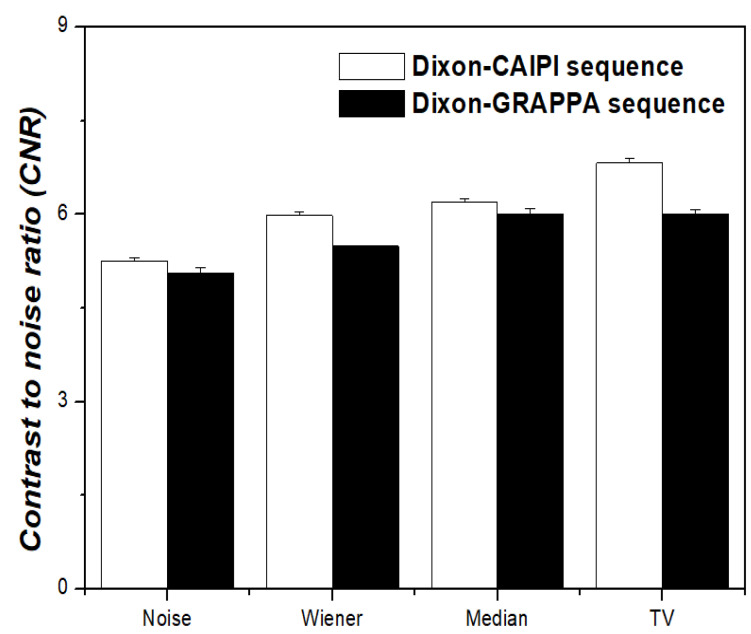
Contrast-to-noise ratio (CNR) according to noise-reduction techniques between MR-AC_Dixon-CAIPI_-based and MR-AC_Dixon-GRAPPA_-based PET images.

**Figure 6:**
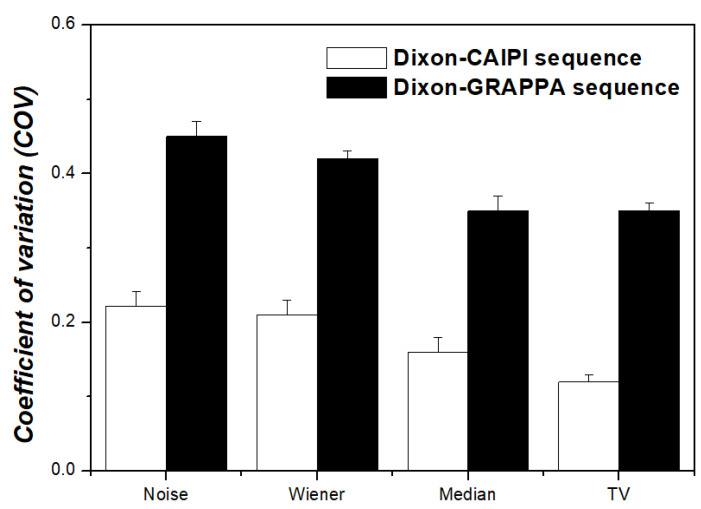
Coefficient of variation (COV) according to noise-reduction techniques between MR-AC_Dixon-CAIPI_-based and MR-AC_Dixon-GRAPPA_-based PET images.

The CNR and COV results are shown in Figure 5 and Figure 6, respectively. When the MR-AC_Dixon-CAIPI_-based PET images were compared with the TV algorithm and noise, Wiener filter, and median filter regarding the CNR, the PET images with the TV algorithm showed values that were approximately 23.0%, 12.3%, and 9.1% higher than those with noise, Wiener filter, and median filter, respectively. When the MR-AC_Dixon-GRAPPA_-based PET images were compared with the TV algorithm and noise, Wiener filter, and median filter with respect to the CNR result, the PET image with the TV algorithm showed values that were approximately 15.8% and 8.7% higher than images with noise and the Wiener filter, respectively. When comparing the images with the TV algorithm and median filter, there was no significant difference with respect to the CNR results in the MR-AC_Dixon-GRAPPA_-based PET images. In addition, when the CNR results of the MR-AC_Dixon-CAIPI_-and MR-AC_Dixon-GRAPPA_-based PET images were compared, the MR-AC_Dixon-CAIPI_-based PET images showed values that were approximately 8.2%, 3.1%, and 11.8% higher than that of PET images with the Wiener filter, median filter, and TV algorithm, respectively. In summary, the MR-AC_Dixon-CAIPI_-based PET image had a value that was approximately 6.7% higher than that of the MR-AC_Dixon-GRAPPA_-based PET image with respect to the CNR result. Concerning the COV result, the MR-AC_Dixon-CAIPI_ based PET images were approximately 27.9% and 23.8% lower than images with noise and Wiener filter, respectively. In addition, the COV values for MR-AC_Dixon-GRAPPA_-based PET images were approximately 22.2% and 16.7% lower than images with noise and Wiener filter, respectively. There was no significant difference with respect to the MR-AC_Dixon-CAIPI_-and MR-AC_Dixon-GRAPPA_-based PET images and the COV results between the median filter and TV algorithm. When the MR-AC_Dixon-CAIPI_-and MR-AC_Dixon-GRAPPA_-based PET phantom images were compared for the COV results, the MR-AC_Dixon-CAIPI_ based PET images had values that were approximately 50.6%, 50.0%, 54.3%, and 54.3% lower than images with noise, Wiener filter, median filter, and TV algorithm, respectively. In summary, the COV value of the MR-AC_Dixon-CAIPI_-based PET image was approximately 52.3% lower than that of the MR-AC_Dixon-GRAPPA_-based PET phantom image.

In clinical PET/MR, the time for image acquisition is related to image quality. The narrow bore size (~60 cm) compared with that of PET/CT (~70 cm) leads to motion artifacts and is uncomfortable for patients. In general, PET/MR acquisition time is longer than that of PET/CT due to a scan procedure of only a PET image, MR-AC pulse sequences, and then only an MR image. Among these procedures, the MR-AC pulse sequence can control the acquisition time using various sequences. The MR-AC_Dixon-CAIPI_ pulse sequence which takes a short time for acquisition, is an important factor, because a long acquisition time can affect artifacts in a PET/MR breath-hold scan. Therefore, the MR-AC_Dixon-CAIPI_ pulse sequence is more useful than the MR-AC_Dixon-GRAPPA_ pulse sequence in terms of time resolution. In addition, the fusion images from PET and MR were used to evaluate functional and anatomy information using various MR pulse sequences for patients. Compared with MR CAIPI and GRAPPA pulse sequences in the clinical images, when the MR CAIPI pulse sequence was used, the image quality was improved, according to Wright K. L. et al. [38].

Research on the improvement of image quality in PET/MR has been previously performed for attenuation correction based on MR pulse sequences. Grafe et al. indicated that an improved MR-AC pulse sequence that is divided into five compartments (background, fat, lungs, soft tissue, and bone) can improve image quality by adding bone segmentation, compared with the conventional attenuation correction method [39]. In addition, we need to apply the noise-reduction algorithm, which is broadly applied to reduce noise in medical images. In this study, we confirmed the efficiency of the TV algorithm for noise reduction in PET/MR images. The application of the noise-reduction algorithm can offer accurate diagnostic information, except for unnecessary radiation exposure. Therefore, the noise-reduction algorithm and MR-AC pulse sequence are essential processes for improving the PET/MR image quality. Based on our results, the application of the noise-reduction algorithm can offer accurate diagnostic information while preventing unnecessary radiation exposure and patient discomfort.

In addition, we plan to follow up on two aspects in future studies. First, we plan to compare the image quality using various noise-reduction algorithms such as the median modified Wiener filter, the fast nonlocal means algorithm, and deep-learning techniques in nuclear medicine images. Second, we will attempt to evaluate image performance in PET/MR according to fluid material in the Jaszczak phantom, such as NiSO_4_ + NaCl and only NiSO_4_, instead of water, which is widely used to perform the phantom experiment using the Jaszczak phantom with the gamma camera or PET/computed tomography. Because water interferes with the generation of MR-AC PET images, the new fluid material should be used for the acquisition of MR-AC PET images. For this reason, Ziegler et al. reported that the use of an alternative fluid material, such as NiSO_4_ + NaCl or only NiSO_4_, can lead to obtaining MR-AC PET images [40]. In addition, Park et al. suggested that NaCl fluid is as effective as NiSO_4_ + NaCl fluid, without obstructing MR-AC PET images [41].

## 4. Conclusions

In this study, we confirmed the effectiveness of the TV algorithm for noise reduction using MR-AC_Dixon-CAIPI_- and MR-AC_Dixon-GRAPPA_-based PET images compared with conventional noise-reduction methods in PET/MR using the Jaszczak phantom with the ^18^F radioisotope in an integrated PET/MR scanner. The CNR and COV results demonstrated that the application of the TV algorithm was capable of improving the PET image quality. In conclusion, the TV algorithm is applicable to MR pulse sequence-based PET images in PET/MR with respect to noise reduction, especially MR-AC_Dixon-CAIPI_-based PET images, which are acceptable using the TV noise-reduction algorithm.

## Data Availability

Not applicable.
